# A Novel KGP Algorithm for Improving INS/GPS Integrated Navigation Positioning Accuracy

**DOI:** 10.3390/s19071623

**Published:** 2019-04-04

**Authors:** Huibing Zhang, Tong Li, Lihua Yin, Dingke Liu, Ya Zhou, Jingwei Zhang, Fang Pan

**Affiliations:** 1 Guangxi Key Laboratory of Trusted Software, Guilin University of Electronic Technology, Guilin 541004, China; zhanghuibing@guet.edu.cn (H.Z.); 1603303018@mails.guet.edu.cn (T.L.); liudingke00@163.com (D.L.); ccyzhou@guet.edu.cn (Y.Z.); gtzjw@guet.edu.cn (J.Z.); 2 Cyberspace Institute of Advanced Technology, Guangzhou University, Guangzhou 510006, China; 3 Teaching Affairs Office, Guangxi Normal University, Guilin 541004, China; panfang@mailbox.gxnu.edu.cn

**Keywords:** INS/GPS integrated navigation, data fusion, Gradient Boosting Decision Tree

## Abstract

The fusion of multi-source sensor data is an effective method for improving the accuracy of vehicle navigation. The generalization abilities of neural-network-based inertial devices and GPS integrated navigation systems weaken as the nonlinearity in the system increases, resulting in decreased positioning accuracy. Therefore, a KF-GDBT-PSO (Kalman Filter-Gradient Boosting Decision Tree-Particle Swarm Optimization, KGP) data fusion method was proposed in this work. This method establishes an Inertial Navigation System (INS) error compensation model by integrating Kalman Filter (KF) and Gradient Boosting Decision Tree (GBDT). To improve the prediction accuracy of the GBDT, we optimized the learning algorithm and the fitness parameter using Particle Swarm Optimization (PSO). When the GPS signal was stable, the KGP method was used to solve the nonlinearity issue between the vehicle feature and positioning data. When the GPS signal was unstable, the training model was used to correct the positioning error for the INS, thereby improving the positioning accuracy and continuity. The experimental results show that our method increased the positioning accuracy by 28.20–59.89% compared with the multi-layer perceptual neural network and random forest regression.

## 1. Introduction

With the development of sensor technology, context-aware vehicles (e.g., location services and automatic driving) are becoming increasingly popular. However, these applications require high context perception accuracy, especially in assisted and automatic driving, which has increasingly high requirements for the continuity, reliability, and accuracy of vehicle positioning. The positioning performance of a single Global Positioning System (GPS) may be decreased by various factors, such as occlusion and interference when driving on urban roads [1,2]. Thus, it is difficult to meet the needs of the Internet of Vehicles. However, multi-source sensor fusion, e.g., Inertial Navigation System (INS) and GPS integrated navigation systems, can effectively solve these problems [3,4].

For the data fusion problem in integrated navigation, Kalman Filter (KF) is the existing optimal trajectory estimation method, which solves the problem of tedious calculation caused by the weak nonlinear ability. Particle Filter (PF) is considered a benchmark of the filtering method for predicting vehicle position, but the large number of particles required by PF leads the algorithm computationally expensive. In integrated navigation, when the GPS signal is interrupted [5,6], the positioning error in the Inertial Measurement Unit (IMU) accumulates over time, decreasing the overall performance of the integrated navigation [7,8,9]. To improve the positioning performance during GPS signal interruption, Artificial Neural Networks (ANNs) have been introduced into the INS/GPS integrated navigation system, e.g., Multilayer Perceptron Neural Networks (MLPNNs) [10,11], Radial Basis Function Neural Networks (RBFNNs) [12,13,14], Long Short Term Memory Recurrent Neural (LSTM-RNNs) [15], networks and adaptive Neuron-Fuzzy Inference Systems (ANFISs) [16,17]. The main idea is to train the relationship between the vehicle feature data and INS errors through ANNs when the GPS signal is available. When the GPS signals are available, the current or latest training model is used to predict the positioning data. This method effectively reduces the positioning error and ensures positioning continuity. Since the ANN is trained completely using input data, its generalization ability is limited when the vehicle state data during training is different from that during prediction. To solve this problem, in one study [18], ensemble learning was included in the INS/GPS integrated navigation system, which effectively improved the generalization ability. Although the Least Squares Boosting and Bagging algorithms proposed in that study [18] could improve the positioning accuracy, the errors of the INS internal sensor (e.g., steering deviation, running deviation, and scale factor drift) increased the nonlinear complexity of the relationship between the input and output data. The model was weak at recognizing feature variables, resulting in unsatisfactory predictions of positioning. In addition, the sensor’s errors accumulated over time. When the GPS signal loss was over 5 min, the prediction accuracy of the ensemble learning scheme began to decrease gradually [18].

To solve the above-mentioned problems, a Kalman Filter-Gradient Boosting Decision Tree-Particle Swarm Optimization (KF-GBDT-PSO, henceforth denoted KGP) data fusion method is proposed herein [19], which consists of two consecutive phases: Training and prediction. In the training phase, the KGP prediction model can compensate for the INS positioning error through the relationship between vehicle feature data and KF estimations of the positioning error [20,21]. In the prediction phase (during GPS signal loss), the trained model immediately predicts the positioning data. Compared with ANNs, the predicted values of the GBDT are obtained through accumulating the residuals of multiple trees. Due to its advantage of reducing model deviations, the Gradient Boosting Decision Tree (GBDT) provides a better generalization ability with better accuracy. Additionally, selecting regression parameters can be challenging. Thus, Particle Swarm Optimization (PSO) is introduced in the training phrase to select the optimal parameters for GBDT [22]. The KGP not only could extract nonlinear vehicle feature data in parking and driving states using the addition model and the forward distribution algorithm but could also use the Huber loss function to eliminate the location outliers collected due to road complexity. By flexibly covering various types of variables, the error rate of the integrated navigation system was effectively reduced [23], increasing the prediction accuracy of the positioning.

The remainder of this paper is organized as follows. In Section 2, an overview of the GBDT and PSO theories is provided. In Section 3, the integration scheme for the KGP method is introduced and discussed in detail. The experimental results are discussed in Section 4. Conclusions are presented in Section 5.

## 2. Improved Methods

### 2.1. GBDT Regression Algorithm

The GBDT consists of a gradient boosting and regression decision tree. The decision tree uses a Classification and Regression Tree (CART) as the base learner, which makes predictions quickly but can easily overfit [24]. Gradient boosting can improve the model performance and reduce the fitting ability of the decision tree by changing the weight of the sample [19]. The gradient boosting method and the decision tree learning algorithm complement each other, increasing the overall performance.

When training the model, we input the training sample $D={\left\{\left(x_{i},y_{i}\right)\right\}}_{i=1}^{n}$, where $x_{i}$ is the feature vector of the ith input sample, $y_{i}$ is the sample tag of $x_{i}$, and *n* is the number of sample feature. Next, we initialized the learner as follows:

(1) $$f_{0}\left(x\right)=arg\;\underset{c}{\min}\sum_{i=1}^{N}L\left(y_{i},c\right).$$

We used a strong learner $f\left(x_{i}\right)$ and sample tag $y_{i}$ to construct the loss function $L\left(y_{i},f\left(x_{i}\right)\right)$. 

Freidman [19] defined the loss function as:

(2) $$L\left(y_{i},f\left(x_{i}\right)\right)=\overset{N}{\underset{i=1}{\sum }}\frac{1}{2}{\left[y_{i}-f\left(x_{i}\right)\right]}^{2}.$$

**Figure 1:**
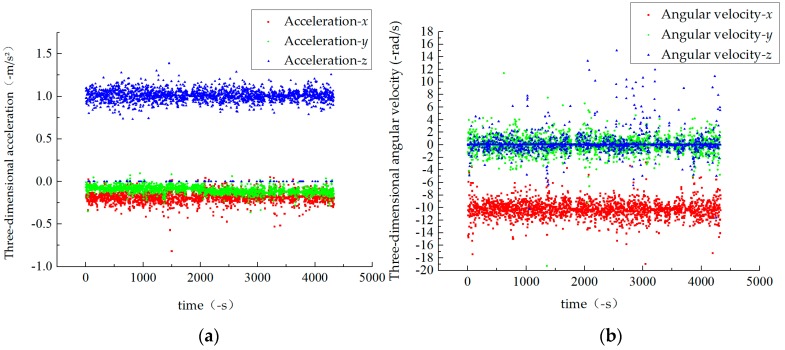
Scatter plot of reference data: (**a**) acceleration and (**b**) angular velocity.

By observing the data characteristics of the vehicle, we found that the gyroscope and accelerometer contained in the IMU are in the process of measuring the real road, and the data outliers will inevitably be collected due to the interference of the urban environment. In order to visually observe the measured values, the data in the three directions of acceleration and angular velocity are displayed by a scatter plot (Figure 1); it is well known that the anomaly measurement information increases the complexity of the system and largely affects the prediction accuracy of the navigation model.

Therefore, in this study, the loss function is defined as follows based on real data:

(3) $$L\left(y_{i},f\left(x_{i}\right)\right)=\{\begin{array}{c} \frac{1}{2}{\left[y_{i}-f\left(x_{i}\right)\right]}^{2}\;\;\left|y_{i}-f\left(x_{i}\right)\right|<\delta \\ \delta \left|y_{i}-f\left(x_{i}\right)\right|-\frac{\delta }{2}\;\left|y_{i}-f\left(x_{i}\right)\right|<\delta . \end{array}$$

Here, δ is measured by quantile, and the value is 0.9. The Huber loss function uses absolute loss for anomaly vehicle data. For normal data, we used the mean square error to effectively exclude inappropriate data. This not only maintains the continuity of the loss function, but also has better robustness to outliers.

In order to ensure the continuous decline of the loss function, the negative gradient of the loss function is used in the iteration to calculate the approximate value of the current model residuals. For the *m*^th^ iteration, the negative gradient is defined as:

(4) $$r_{im}(y_{i},f\left(x_{i}\right)=\{\begin{array}{c} y_{i}-f\left(x_{i}\right)\;\;\left|y_{i}-f\left(x_{i}\right)\right|\leq \delta \\ \delta sign\left(y_{i}-f\left(x_{i}\right)\right)\;\left|y_{i}-f\left(x_{i}\right)\right|\geq \delta . \end{array}$$

We fit ($x_{i},r_{im}$) to obtain t CART regression trees. Each tree is represented by $h_{t}\left(x\right)$, and its corresponding leaf node area is ${\left\{R_{i}\right\}}_{1}^{J}$, where J represents the number of leaf nodes in the regression tree. The linear search is used to estimate values of each leaf node region of the regressed trees, which can minimize the loss function.

The traditional GBDT model does not introduce regularization, so it is easy to overfit complex data. To prevent this phenomenon, a regularization term (5) is introduced in the loss function to penalize the number of leaf nodes in each CART tree, which is equivalent to pruning the regression tree during the training process.

(5) $$\Omega f=\gamma T+\frac{1}{2}\lambda \|\omega \|$$

Initially, the learning weights of the samples were the same. As the regression tree grew with iterations completed, samples’ weights were updated. Samples having low prediction accuracies were assigned larger weights, and high accuracy samples were assigned smaller weights. The weights were defined as follows:

(6) $$\rho_{m}=\arg\underset{\rho }{\min}\overset{N}{\underset{i=1}{\sum }}L\left(y_{i},f_{m-1}\left(x_{i}\right)+\rho_{m}h_{m}\left(x\right)\right).$$

Through updating the residuals of the regression trees, we obtained a strong learner model as follows:

(7) $$f_{m}x_{i}=f_{m-1}\left(x_{i}\right)+\rho_{m}h_{m}\left(x\right).$$

Therefore, the final GBDT model is

(8) $$F\left(x\right)=F_{0}\left(x\right)+v\overset{N}{\underset{i=1}{\sum }}\rho_{m}h_{m}\left(x\right),$$

where v(0<v<1) is the learning rate that determines the iteration of GBDT.

### 2.2. PSO Algorithm

The regression parameters in the GBDT determine the prediction accuracy of the model. Parameter selection requires extensive experience or large-scale searching. We introduced PSO into the GBDT to search for high-quality parameters [25], as it was easy to implement and improved the global optimization and convergence velocity.

In the PSO algorithm, each particle represents a parameter. The model’s particle dimension is θ=(v,m,l,d), where v,m,l,d respectively represent the learning rate, the number of iterations, the minimum number of leaves, and the maximum depth of the regression tree in the GBDT.

In four-dimensional space, we generate a set of position vectors $X_{\theta }$ and flight velocity vectors $V_{\theta }$. Each particle represents an adaptive value assigned by the objective function fitness (·). To obtain the optimal objective values of the regression parameters, the particles’ positions and velocity s are updated with reference to their two current extreme values as follows [26]:

(9) $$V_{\theta }\left(t\right)=\omega V_{\theta }\left(t-1\right)+c_{1}r_{1}\left(P_{best}^{\theta }-X_{\theta }\left(t-1\right)\right)+c_{2}r_{2}\left(G_{best}^{\theta }-X_{\theta }\left(t-1\right)\right),$$

(10) $$X_{\theta }\left(t\right)=X_{\theta }\left(t-1\right)+V_{\theta }\left(t\right).$$

where ω is a negative inertia factor; $c_{1}$ and $c_{2}$ are the particle learning rate and global learning rate, respectively; $r_{1}$ and $r_{2}$ are random numbers between 0 and 1; and $P_{best}^{i}$ and $G_{best}^{i}$ represent the particle and global best locations, respectively.

The algorithm continuously updates $P_{best}^{i}$ and $G_{best}^{i}$ based on the calculated particle position, velocity, and adaptive function values. The regression parameter’s optimal value is determined when the maximum number of iterations or the accuracy requirement is reached.

## 3. Integration Scheme of KGP

An “East-North-Up” (ENU) geographic coordinate system was used in this work as the vehicle navigation coordinate system. The system’s origin is located at the vehicle’s center of mass, where the x and y axes are in tangential directions of the local meridian and parallel, respectively, and the z axis is in the vertical direction.

**Figure 2:**
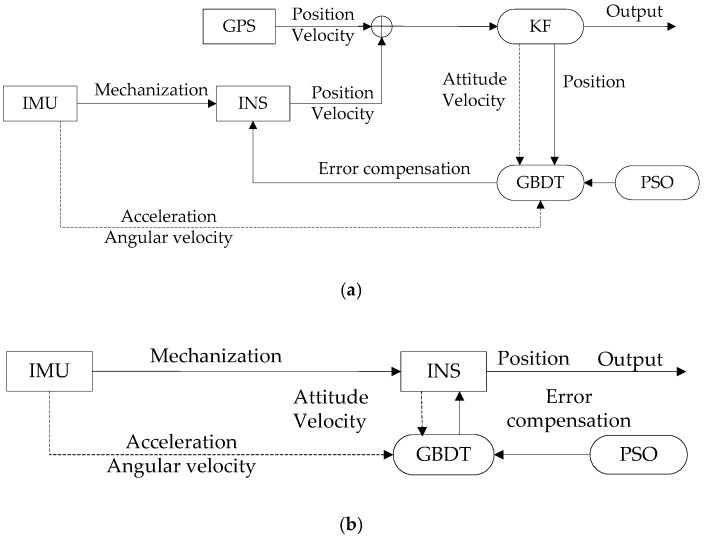
KGP prediction model diagram: (**a**) training mode and (**b**) prediction mode.

Most vehicles run closely to the ground; the horizontal plane error was used in this study as an indicator to measure the performance of the vehicle navigation system during GPS signal interruptions. Figure 2 describes the INS/GPS integrated navigation system based on KGP.

The KGP includes two phases: training and prediction. We input vehicle data in the east-north direction (including velocity, posture, and the outputs of the gyroscope and accelerometer) to the GBDT model. The GBDT model output estimated the two-dimensional position errors of the KF filter. The KGP predictive model’s input/output formulas are defined as follows:

(11) $$\begin{array}{ll} \mathrm{Input}: & X\left(t-s\right)=\left[V_{t-s}^{e}\;V_{t-s}^{n}\;\Psi_{t-s}^{e}\;\Psi_{t-s}^{n}\;w_{t-s}\;a_{t-s}\right]. \end{array}$$

(12) $$\begin{array}{ll} \mathrm{Output}: & \Delta Y_{t}=\left[\Delta p_{t}^{e}\;\Delta p_{t}^{n}\right]=\left[p_{t}^{e}-p_{t-s}^{e}\;p_{t}^{n}-p_{t-s}^{n}\right]. \end{array}$$

The subscript *s* indicates the data processing time interval; $\left[V_{t-s}^{e}\;V_{t-s}^{n}\right]$ and $\left[\Psi_{t-s}^{e}\;\Psi_{t-s}^{n}\right]$ are the velocity and angles in the east-north direction, respectively; *w* and *a* respectively represent the angular velocity from the gyroscope and the acceleration from the accelerometer; and $p_{t}^{e}$ and $p_{t}^{n}$ respectively represent the east and north positions after the latitude and longitude conversion.

When the GPS signal is normal, the system is in training mode; its function diagram is shown in Figure 2a. The system builds the KGP model using relationships between the temporary input/output variables:

(13) $$P\left(x\right)=P_{0}\left(x\right)+v\overset{N}{\underset{i=1}{\sum }}\rho_{t}h_{t}\left(x\right).$$

The above formula is used to calculate vehicle data, such as the velocity, posture, and relationship between the IMU output and the KF estimation error through fitting.

To improve the quality of the GBDT regression parameters, PSO was adopted. The $c_{1},c_{2}$, and ω parameter values are shown in Table 1 [27]. The adaptive function was defined as the standard mean squared error (MSE):

(14) $$Fit\left(t\right)=\frac{1}{N}\overset{N}{\underset{d=1}{\sum }}{\left(y_{s}-{\hat{y}}_{d}\right)}^{2}.$$

In the above equation, Fit(t) represents the fitness value of the t-dimensional particle, $y_{s}$ is the sample output value, and ${\hat{y}}_{d}$ is the sample prediction value. The optimal value can be obtained by continuously iterating the GBDT regression parameters. The values are shown in Table 2.

When the GPS signal is interrupted, the KGP switches to prediction mode, as shown in Figure 2b. At this time, only the INS is operating in the integrated navigation system. The system sends the combined prediction estimation error back to the INS and generates predicted positioning data while correcting the INS position error in real time.

## 4. Results and Discussion

Two experiments were conducted. In Experiment 1, the generalization ability of the training model to the INS error under different driving states of the vehicle was investigated. In Experiment 2, the KGP model’s ability to compensate for the INS error in its positioning predictions was tested. We also compared the results from the KGP with that from the MLPNN and random forest regression (RFR) [28,29].

We used the Chery eQ model to build the experimental platform, with the built-in GNSS receiver model MC20 and MG10 inertial navigation system as the hardware measurement equipment to collect reference data. The GPS position measurement accuracy was less than 2 m; the output frequency was 1 Hz; and the velocity measurement accuracy was 0.185 m/s, updated at 1 Hz. The output frequency of the INS inertial measurement unit was 1 Hz, and the sample IMU was used to acquire the linear and angular velocity of the vehicle at 1 Hz. The study was conducted in Guilin City, Guangxi Province, China, under five different road conditions: Straight, curved, sloped, downhill, and at an intersection with acceleration and deceleration. 

### 4.1. Model Generalization Ability

**Figure 3:**
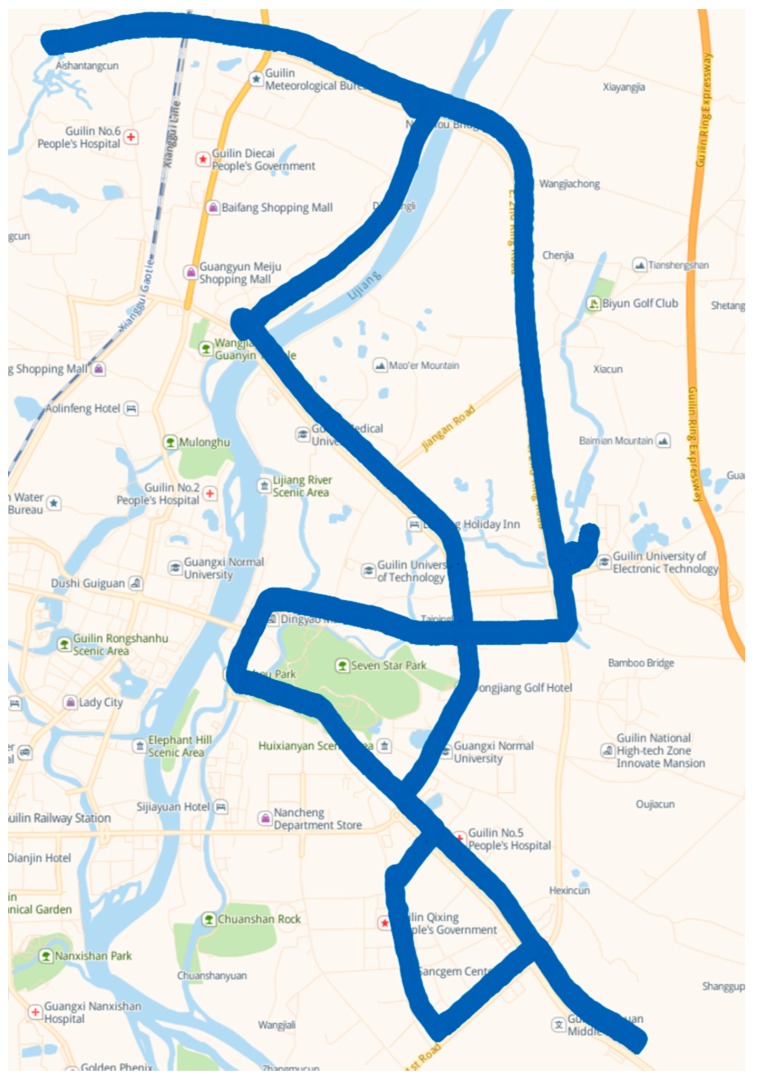
Experiment 1: Vehicle driving trajectory.

**Figure 4:**
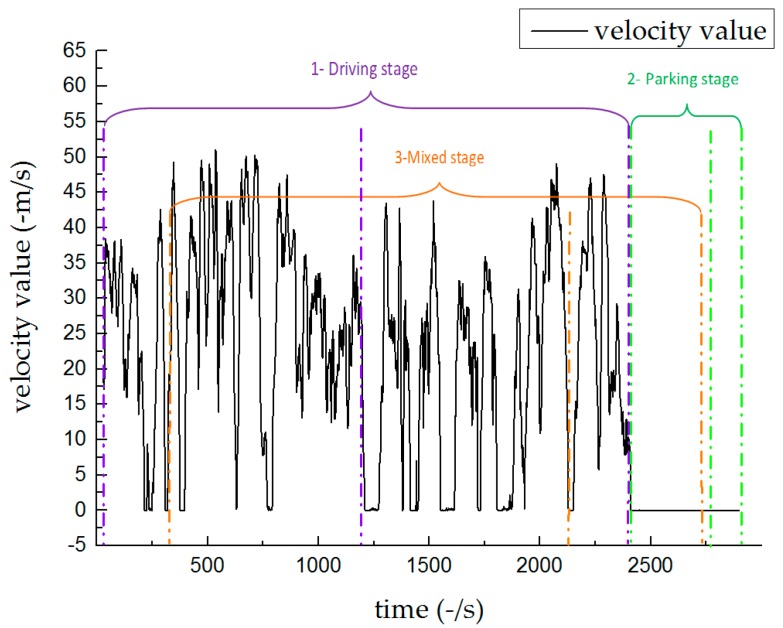
Experiment 1: Vehicle driving velocity.

The vehicle was tested on the road, shown in Figure 3. The tests included three types: A normal driving test (e.g., stops at traffic lights, corner turns, acceleration, and deceleration), a parking test (with only engine working), and a combined test (normal driving and subsequent parking). Figure 4 shows the vehicle’s velocity s during the three tests over 48 min. Table 3 lists the time frames for the tests.

#### 4.1.1. Results of the Normal Driving Test

**Figure 5:**
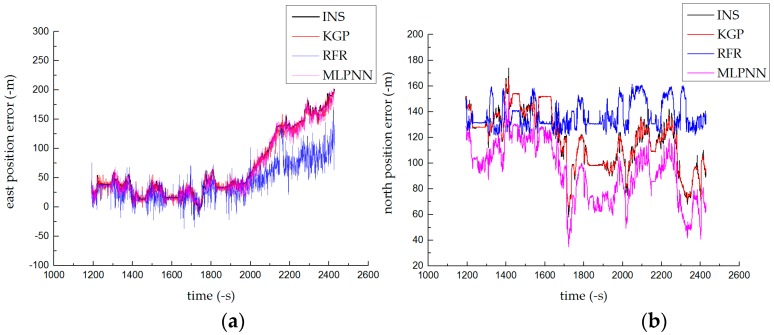
Prediction results of the normal driving test: (**a**) north position error and (**b**) east position error.

Figure 5 depicts three algorithms that predicted the positioning error of the INS in the east-north direction. The RFR algorithm obtained better predictions [22] in shorter times. The prediction error of the RFR gradually decreased after the GPS signal was interrupted for over 5 min. Since we use the squared difference as the loss function, the MLPNN algorithm yielded better predictions in the latter half. The KGP algorithm was better than other algorithms in that it maintained good generalization ability for 20 min, reduced the positioning error in the north direction from 142 to 1.13 m, and reduced the positioning error to 0.051 m in the east direction.

As shown in the figure, there was a significant cumulative error in the east direction, and the positioning error in the north direction was relatively stable. This is because the KGP method was set up with a positioning fix. Errors were compensated immediately when the parking time exceeded 5 s. For example, the positioning fix began working if the vehicle keeps moving forward at traffic light. The east direction corresponds to the y-axis of the inertial coordinate system, and the vehicle frequently turns and changes lanes after restarting at traffic lights. Therefore, the positional compensation effect of KGP in the east direction was not significant. 

#### 4.1.2. Results of the Parking Test

**Figure 6:**
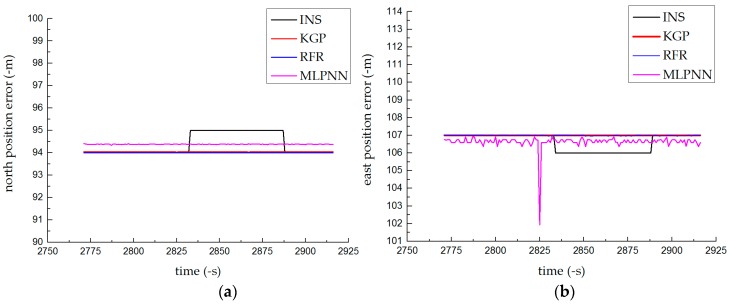
Prediction results of the parking test: (**a**) north position error and (**b**) east position error.

The positioning error shown in Figure 6 exhibited small fluctuations. Since the engine was still running, vehicle shaking could cause small errors in the INS. The prediction accuracies of the three methods were not much different, and the overall performance was better than that of INS alone. The prediction results of the GBDT and RFR were better than that of MLPNN because the initial weights and thresholds of the MLPNN were random, resulting in different results for each calculation.

#### 4.1.3. Results of the Combined Test

**Figure 7:**
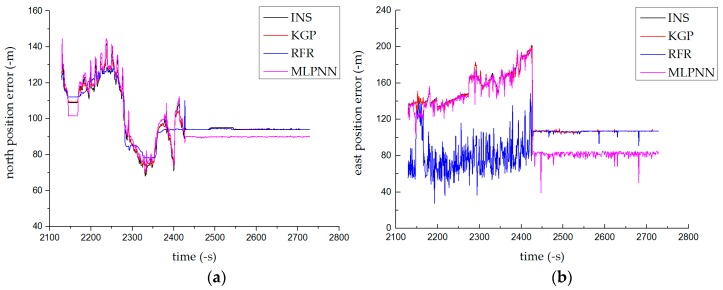
Prediction results of the combined test: (**a**) north position error and (**b**) east position error.

The combined test used the training model in the driving phase to predict INS errors in driving (motion) and parking (stationary) states. As shown in Figure 7, the RFR fluctuated greatly in the driving state, and the prediction accuracy was far lower than that in the parking state. When the vehicle’s state in the training phase was inconsistent with that in the prediction phase, the generalization ability of the MLPNN was greatly weakened; the prediction was good only in the driving state. As a result, compared with other methods, the KGP algorithm had the highest positioning accuracy and could fit the trajectory curve well. The prediction was accurate and stable under various states. Table 4 shows the INS absolute error maxima in the driving and parking states in Test 3. 

Compared with the predicted results of the normal driving test, with increased amounts of training data, the combined test could obtain more accurate positioning in a shorter time than the normal driving test. 

### 4.2. Model Validity

**Figure 8:**
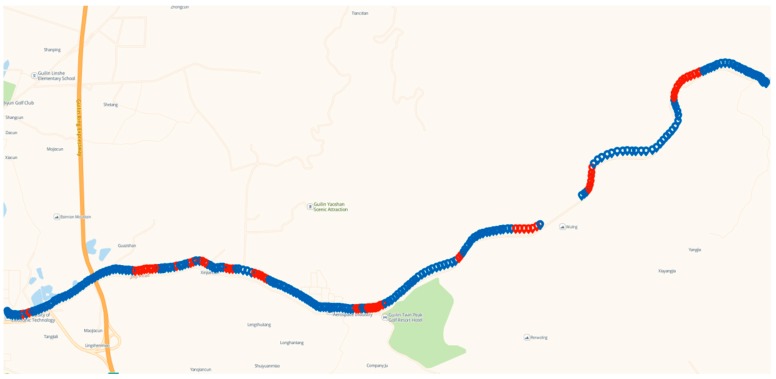
Experiment 2: Vehicle trajectory.

**Figure 9:**
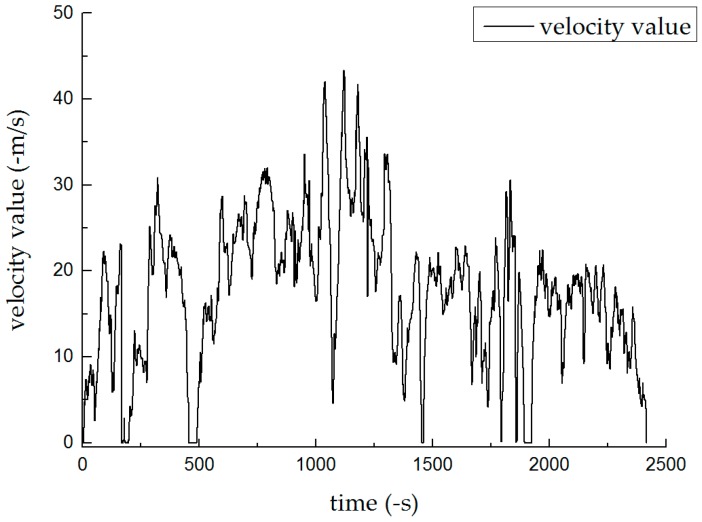
Experiment 2: Vehicle Speed.

The prediction models for the experiments described in Section 4.1 were applied to different roads (Figure 8). The system switched between training and prediction modes based on the actual conditions of the road, and the positioning prediction was divided into three phases. The blue area indicates good GPS navigation. The red area represents an occlusion test when the GPS signal was unstable and the accuracy was lowered due to high-rise buildings on both sides of the road. The blank area represents a tunnel test in which the GPS lost its lock due to the vehicle passing through a tunnel. Figure 9 shows the vehicle’s velocity during 40 min of travel.

**Figure 10:**
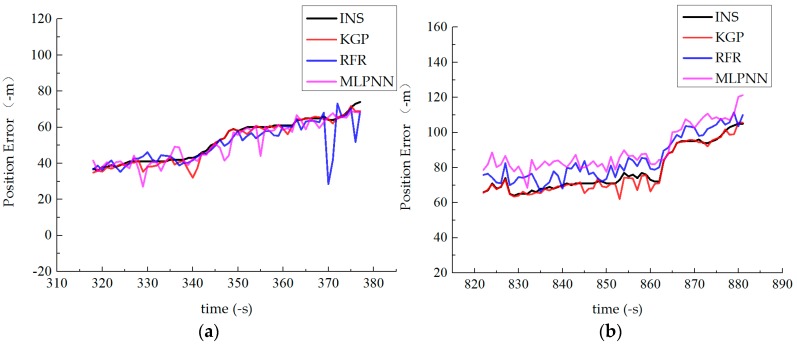

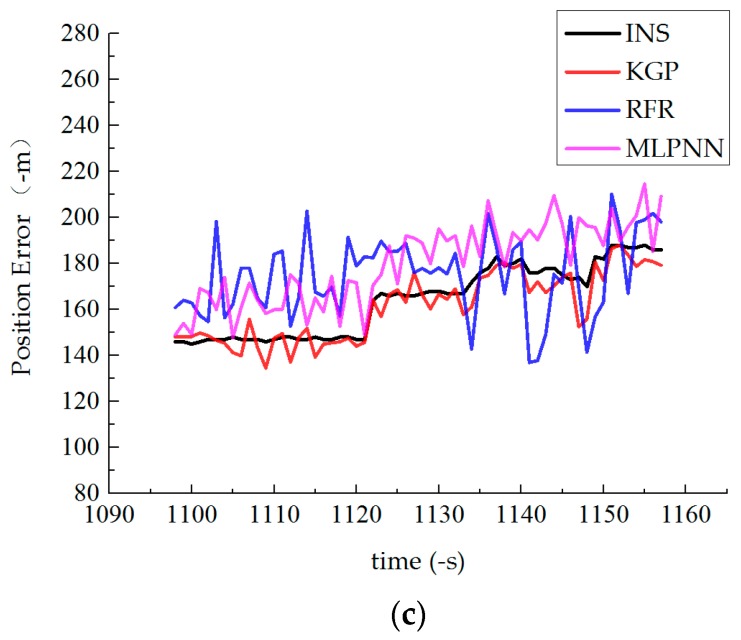
Comparison results of different algorithms in position errors: (**a**) phase 1, (**b**) phase 2 and (**c**) phase 3.

Figure 10 depicts the final positioning error of these algorithms in three phases.

In phase 1, when the vehicle was driving on the city street which was straight and narrow for more than 5 min, the GPS signal was unstable. When the road was crowded, it was accompanied by frequent acceleration and deceleration, which enhanced the nonlinear relationship between input and output. Therefore, the prediction accuracy of MLPNN was low in the initial stage, RFR and KGP could fit the error curve well. But when the time was longer than 60 s, the prediction accuracy of RFR decreased, and KGP maintained strong generalization ability.

In phase 2, the vehicle entered the tunnel, and the INS error accumulation speed increased due to the complete loss of signals in the tunnel. It could be seen from Figure 10b that the prediction performance of KGP was higher than that of RFR and MLPNN algorithms. The maximum error was only 11.03 m, and the prediction result was stable.

In phase 3, the vehicle was driving on a curved road. The driving velocity and direction will change greatly with the curve trajectory, resulting in serious system cumulative error and less data set in a short time. Therefore, the prediction accuracy of the three methods was far less than that of phase 1 and phase 2, and there was a slight oscillation. But KGP still had obvious advantages compared with other methods, which was attributed to the improved KGP that could find out the relationship between the input and output, make up for the inherent error of the sensor, effectively model the position errors.

To fully evaluate the effectiveness of the system’s positioning data, we used the Root Mean Square Error (RMSE) to compare the predicted performance of the three methods. The RMSE is defined as follows (15):

(15) $$RMSE=\sqrt{\frac{\sum_{i=1}^{T}{\left({\hat{y}}_{p}-y_{p}\right)}^{2}}{T}},$$

where T is the GPS signal interruption time and ${\hat{y}}_{p}$ and $y_{p}$ represent the predicted value and the actual output value, respectively.

Table 5 shows the RMSEs of different prediction algorithms. Compared with the RFR and MLPNN, the predicted result of the KGP algorithm had less error, and the predicted positioning data was closer to the reference data.

Although the RFR and GBDT are both ensemble learning algorithms, the RFR continues to reduce the variance of the data set during the training phrases, while the GBDT improves the accuracy by reducing bias. Therefore, the RFR prediction produced larger errors, while the KGP prediction yielded better agreement with the data collected by the vehicle.

## 5. Conclusions 

The KGP model can better meet the needs of accurate positioning during GPS signal interruption. The GBDT algorithm was integrated based on KF, and the regression parameters of the GBDT were optimized by the PSO algorithm to obtain a better prediction model. The road test showed that the generalization ability of the GBDT algorithm was stronger than that of a single algorithm in the integrated learning. The KGP model could effectively compensate for the cumulative error of the INS and correct the position data during GPS failures. The prediction remained accurate during a 20 min period. Compared with the existing methods, the positioning accuracy of our navigation solution was 28.20–59.89% higher than those of the MLPNN and RFR.

## Figures and Tables

**Table 1:** Parameters for PSO.

Parameter	Value
Particle factor ($c_{1}$)	0.103
Population factor ($c_{2}$)	2.897
Inertia weight (ω)	0.6

**Table 2:** Values of parameters for GBDT.

Parameter	Value
Learning rate (*ν*)	0.05
Number of iterations (*m*)	514
Minimum number of leaf (*l*)	2
Max depth(*d*)	8

**Table 3:** Time allocations of different independent stage.

	GPS On (s)	GPS Loss(s)	Total Time (s)
Driving	29–1192	1192–2429	2400
Parking	2429–2771	2771–2909	480
Combined	328–2129	2129–2728	2400

**Table:** Driving state.

Position Error Value	INS		KGP (m)		RFR (m)		MLPNN (m)	
	North	East	North	East	North	East	North	East
Maximum (m)	142	201	138.66	201.67	128.64	151.43	144.52	198.82
Minimum (m)	68	107	68.97	106.50	78.22	27.55	70.53	81.06
Average (m)	103.78	156.41	103.78	155.45	103.93	79.46	105.51	152.35

**Table:** Parking State.

Position Error Value	INS		KGP (m)		RFR (m)		MLPNN (m)	
	North	East	North	East	North	East	North	East
Maximum (m)	95	107	94.81	109.84	94.70	108.21	90.53	84.93
Minimum (m)	94	106	93.86	103.23	93.99	90.86	89.13	39.21
Average (m)	94.18	106.81	94.16	106.81	94.17	106.62	89.92	81.65

**Table 5:** Comparison results of RMSE with different algorithms.

Phase	KGP	RFR	MLPNN
Outage 1 (60 s)	2.63	6.80	4.63
Outage 2 (60 s)	5.02	7.00	11.88
Outage 3 (60 s)	6.28	22.64	16.03
